# Social Disconnection Among Older Adults Receiving Care in the Emergency Department

**DOI:** 10.5811/westjem.2018.9.38784

**Published:** 2018-10-29

**Authors:** Deepika Kandasamy, Timothy F. Platts-Mills, Manish N. Shah, Kim A. Van Orden, Marian E. Betz

**Affiliations:** * University of Colorado School of Medicine, Department of Emergency Medicine, Aurora, Colorado; † University of North Carolina of Chapel Hill School of Medicine, Department of Emergency Medicine, Chapel Hill, North Carolina; ‡ University of Wisconsin School of Medicine and Public Health, Department of Emergency Medicine, Madison, Wisconsin; § University of Rochester School of Medicine and Dentistry, Department of Psychiatry, Rochester, New York

## Abstract

**Introduction:**

Social disconnection is a public health problem in older adults, as it can lead to decreased quality of life for this population. This study describes the prevalence of social disconnection and patient interest in social resources to address social disconnection among older adults receiving emergency department (ED) care.

**Methods:**

We conducted a cross-sectional survey of community-dwelling older adults (≥65 years) receiving care at two U.S. EDs. We described participant characteristics (demographic, social, and health variables), social disconnection prevalence, and desire for social resources using percentages and 95% confidence intervals. Then, we performed Chi Square tests and logistic regression to determine factors associated with positive screens for social disconnection.

**Results:**

Of 289 participants, 51% were female and the median age was 72 (interquartile range: 69–78). Most (76%) engaged with the community regularly, and 68% reported driving. Regarding social disconnection, a substantial minority of participants reported feeling as if they were burdensome to others (37%); as if they didn’t belong (27%); or that people would be better off if they were gone (15%); 52% reported at least one of these. In separate regression analyses, the perceptions of being a burden or better off if gone were each significantly associated with needing help with routine tasks (odds ratio [OR] [5.87, 5.90]); perceived burden was associated with hospitalization in the prior month (OR [2.09]); and low belonging was associated with not engaging in the community regularly (OR [2.50]), not seeing family regularly (OR [3.82]), and difficulty affording food (OR [2.50]). Regarding potential ED referrals, most participants were interested in transportation options (68%), food assistance (58%), and mental health resources (55%). Participants experiencing difficulties affording food were interested in food and housing assistance (p=.03; p=.01).

**Conclusion:**

Over half of this sample of older ED patients reported feeling socially disconnected. Social and functional health problems are often related and both must be addressed to optimize older ED patient quality of life. Future research should consider the impact of social disconnection on older adults discharged from the ED and work to develop ED services that could refer this population to programs that may decrease social disconnection.

## INTRODUCTION

In 2009, adults aged ≥65 years accounted for 18% of visits to emergency departments (ED) in the United States (U.S.).^1^,^2^ Because hospitalization may negatively impact older patients, providers seek safe discharge plans.^3^ Recent Geriatric ED Guidelines^4^ address older ED patients’ physical needs, but important social health determinants (e.g., social support, food, and housing access) receive less focus.^5^,^6^

Social connection refers to how individuals connect with others, comprising both objective (e.g., number of family members seen each week, amount of time spent with others) and subjective (e.g., loneliness, feelings of burdensomeness, feeling like one belongs in relationships) connections.^7^ Social disconnection may increase health risks for older adults.^8^,^9^ Affecting ~43% of this population,^10^ it is associated with negative outcomes such as falls,^11^ cognitive decline,^12^ and mortality.^13^ Two subjective forms of social disconnection are perceptions of burdensomeness, and not “belonging.”^7^ According to the Interpersonal Theory of Suicide (ITS), those feeling burdensome and as if they do not belong (to the point that they feel others would be better off if they were gone) may also experience increased suicidality.^14^–^16^ Older adults with access to resources such as peer companionship, transportation, or food assistance may feel more connected.^17^

Socially disconnected older adults visit EDs more frequently than those feeling socially connected.^18^,^19^ Thus, EDs have opportunities to identify and refer vulnerable older adults to programs to reduce social disconnection. Previous research suggests feasibility of referral interventions and older adult receptiveness to such programs.^20^,^21^

Among older ED patients, we sought to: estimate the prevalence of social disconnection; identify characteristics associated with this factor; and examine social resource needs and desires. Our findings may support ED interventions for connection with community services to enhance well-being.

## METHODS

### Design and Participants

This anonymous, cross-sectional survey took place at two academic EDs (targeting urban and rural populations (65,000 visits yearly) and exclusively urban populations (100,000 visits yearly). Research assistants (RAs) were trained in survey techniques by site principal investigators and they recruited patients 8:00 a.m. to 5:00 p.m., Monday-Friday, from July 2016 – April 2017. RAs identified patients ≥65 years on the ED’s tracking board and asked treating providers to confirm eligibility (medically able to participate and not institutionalized [e.g., prisoners, nursing home residents]). RAs then approached eligible patients, described the survey, and assessed cognitive capacity to participate (could convey the study’s purpose, potential benefits and risk, and voluntary nature). Paper-based surveys were completed independently, or were RA-administered for those with visual or other physical limitations. All approached patients received pamphlets of local resources.

RAs entered surveys into Research Electronic Data Capture (REDCap) for data management.^22^ The Colorado Multiple and University of North Carolina-Chapel Hill institutional review boards approved this project.

### Measures

Questions considered demographic, social, and health characteristics, including portions of the Geriatric Wellness Screening Tool that address social and financial needs.^23^ Three validated Likert-scaled items^24^–^25^ measured social disconnectedness as defined by the ITS. Participants screened positive for *perceived burden* when answering “somewhat” or “very” to “I feel like a burden on the people in my life” and/or to “I feel people would be better off if I was gone.” And they screened positive for *low belonging* when answering “not at all” or “somewhat” to the statement “I feel like I belong.”

### Analysis

We described responses using percentages and 95% confidence intervals (CI), and compared subgroups using chi-square tests. With age and gender included a priori, separate logistic regression models were created considering factors associated with positive screens for (1) perceived burden, (2) low belonging, or (3) better off gone. Then, stepwise modeling identified models with best goodness-of-fit including variables significantly associated (*p*<0.05) with each outcome.

## RESULTS

Of 305 participants, 289 were included in analysis for completing at least two social disconnection questions. The median age was 72 years (interquartile range [69–78] (Table 1); and 51% were female. Most reported regularly interacting with family and friends, engaging with the community, driving vehicles, and easily affording food and to pay bills. Regarding health characteristics and utilization, most had primary care providers and one fourth had experienced hospitalization(s) in the prior month. For Activities of Daily Living, more needed routine task assistance (33%) and assistive equipment (e.g. cane, walker; 41%) than personal care (14%).

### Perceived Social Disconnection

On the social disconnection screen, 37% screened positive for perceived burden, 27% for low belonging, and 15% for feeling better off gone (Table 1). Half (52%) had ≥1 positive social disconnection screens; 7% had three positive screens. Perceived burden related to negative health factors; low belonging related to negative social factors; and feeling better off gone related to health and social factors (Table 1). More non-drivers vs. drivers reported perceived burden (52% vs. 31%, *p*<.000), low belonging (40% vs. 21%, *p*<.001), and feeling better off gone (26% vs. 9%, *p*<.000) (Table 1).

Final regression models showed perceived burden relating to needing routine task assistance (OR [5.9], 95% CI [3.4–10.3] (Table 2), and hospitalization in the preceding month (OR [2.1], 95% CI [1.1–3.8]). Low belonging related to seeing family irregularly (OR [3.8], 95% CI [1.7–3.4]), irregular community engagement (OR [2.5], 95% CI [1.3–4.6]), and difficulty affording food (OR [2.5], 95% CI [1.2–5.1]). Finally, feeling better off gone related to needing routine task assistance (OR [5.9], 95% CI [3.3–10.7]).

### Program Referrals

Many thought referrals for transportation (68%), food assistance (58%), or mental health resources (54%) would be useful (Table 1). Difficulty affording food related to food and housing assistance interest (79%, 95% CI [66–92], *p*=.03; 78%, 95% CI [65–91], *p*=.001). No other notable relationships existed between participant characteristics and social resource desires (not shown). Social disconnection questions and social resource interest were not significantly associated (Table 1).

## DISCUSSION

Social disconnection – measured as perceived burden, low belonging, or feeling others would be better off if [I were] gone – was prevalent in this older ED population. Positive disconnection screens were most associated with hospitalizations in the prior month, needing routine task assistance, and irregular community engagement. Our findings highlight opportunities to improve ED geriatric care, especially for patients discharged home.

Half of participants reported experiencing disconnection, compared to 38% in a primary care sample.^25^ Older adults without social support may have greater ED use because they cannot rely on others for healthcare needs.^18^,^19^ Although social needs may be under-recognized, social and physical problems are often interconnected.^26^ Here, feeling better off gone (which relates to suicidality^27^) was related to needing physical help with routine tasks. In this context, suicidality may increase when physical function and autonomy decrease.^28^ Suicidality is often under-recognized in older adults, including in EDs;^29^ assessing social needs may help with identification and intervention.

Burden factors (perceived burden and feeling better off gone) were related to hospitalization and needing routine task assistance, while low belonging related to irregular community and family contact.^3^,^10^,^30^ Targeting these factors in the ED may improve older adult social outcomes. For example, health factors addressed through ED-based physical and occupational therapy programs may improve function and decrease future hospitalization and readmission;^31^ providing connections to transportation programs^32^ may improve community engagement.

Generally, participants expressed interest in resource referrals. ED teams with social workers and case managers could identify social disconnection and connect patients to social resources (e.g., transportation services, community centers, meal programs).^33^ Because eating is a fundamental context for human social interactions,^34^ addressing food insecurity might provide ways for improving social connectedness.^35^,^36^ In one successful intervention that led to reduced readmissions, nurse practitioners used case-finding systems to identify older adults with unmet medical or social needs and referred them to services.^20^ While such interventions appear feasible, few have been implemented.^21^,^37^ More must be done to test effective health service systems that will increase older adult well-being.^38^

Interestingly, socially disconnected older adults did not desire social resources more than those with social connections. Older adults may not want to burden others with their desires, a reluctance that may extend to social resources. Normalizing discussion about older adult needs may increase access to needed services. In one study, while many older adults wanted services related to their assessed needs, some did not want services that would benefit them and others wanted services misaligned with assessed needs.^39^ Thus, ED-based programs screening for social needs should educate older patients on actual vs. perceived needs and optimal resources and on ways to decrease social disconnection, while also considering the resources that the population feels they may need.^40^

## LIMITATIONS

Results from this convenience sample of English speakers may not generalize to all older ED patients.^41^ The survey did not assess certain factors (e.g., income, race/ethnicity, medical diagnoses); thus, we could not examine how these relate to issues such as social disconnection or social-resources desire.^42^,^43^ Additionally, those with certain neuropathies or disabilities that kept them from participating in this survey may have been under-represented as we reported the prevalence of social disconnectedness.

## CONCLUSION

In this sample of older ED patients, 52% experienced social disconnection and many were interested in ED-referred social resources. The ED may be a site from which such resources could be provided to populations needing social support. Research is needed to understand the impact of social disconnection on recovery after acute illness or injury and to develop and test individualized approaches for decreasing social disconnection in older ED patients.

## Figures and Tables

**Table 1:** Population characteristics and perceived social disconnection (n=289).

Characteristic	Total		Social disconnection positive screen^a^								

			I feel like a burden			I feel like I don’t belong			People would be better off if I was gone		
				
Total	n	%	n	%	CI%	n	%	CI%	n	%	CI%
			
	289	100	109	37.7	-	78	27	-	42	14.8	-
Demographics											
Age (years)											
65–74	98	33.9	44*	44.9	34.9–54.9	29	29.6	20.4–38.8	12	12.2	5.6–18.9
75–84	133	46	39	29.3	21.5–37.2	35	26.3	18.7–33.9	20	15	8.9–21.2
85–92	58	20.1	26	44.8	31.6–58.0	14	24.1	12.8–35.5	10	17.2	7.2–27.3
Gender (Male)	141	48.8	52	36.9	28.8–44.9	36	25.5	18.2–32.8	23	16.3	10.1–22.5
Live with someone	204	70.6	76	37.3	30.6–43.9	50	24.5	18.6–30.5	29	14.2	9.4–19.1
Live in a private home	260	90	95	36.5	30.6–42.4	65*	25	19.7–30.3	36	13.8	9.6–18.1
Employed	55	19	14*	25.5	13.6–37.3	11	20	9.1–30.9	2*	3.6	−1.5–8.7
Volunteer regularly	72	24.9	23	31.9	20.9–43.0	13*	18.1	9.0–27.2	8	11.1	3.7–18.6
Social connections											
Have pet	135	46.7	49	36.3	28.1–44.5	35	25.9	18.4–33.4	14	10.4	5.2–15.6
See family/friends regularly	251	86.9	95	37.8	31.8–43.9	56***	22.3	17.1–27.5	32	12.7	8.6–16.9
Talk to family/ friends regularly	263	91	95	36.1	30.3–42.0	64**	24.3	19.1–29.6	34*	12.9	8.9–17.0
Engage community regularly	220	76.1	78	35.5	29.1–41.8	46***	20.9	15.5–26.3	25	11.4	7.1–15.6
Drive a vehicle	196	67.8	60***	30.6	24.1–37.1	41**	20.9	15.2–26.7	17***	8.7	4.7–12.7
Eat alone regularly	101	34.9	38	37.6	28.0–47.2	28	27.7	18.8–36.6	12	11.9	5.5–18.3
Difficulty affording food	44	15.2	16	36.4	21.6–51.2	21**	47.7	32.4–63.1	10	22.7	9.8–35.6
Difficulty paying bills	66	22.8	29	43.9	31.6–56.2	25*	37.9	25.9–49.9	15*	22.7	12.4–33.1
Health characteristics and utilization											
Has primary care physician	265	91.7	94*	35.5	29.7–41.3	71	26.8	21.4–32.2	37	14	9.8–18.2
Hospitalizations in past month	71	24.6	40***	56.3	44.5–68.2	24	33.8	22.5–45.1	17*	23.9	13.8–34.1
Emergency department (ED) arrival method											
Ambulance	99	34.3	38	38.4	28.6–48.1	30	30.3	21.1–39.5	23*	23.2	14.8–31.7
Drove self	42	14.5	13	31	16.4–45.5	7	16.7	4.9–28.4	38	90.5	81.2–99.7
Family/friend	137	47.4	55	40.1	31.8–48.5	35	25.5	18.2–32.9	17	12.4	6.8–18.0
Other	11	3.8	3	27.3	0.0–58.7	6	54.5	19.5–89.6	1	9.1	−11.2–29.4
Participant disposition (definite/possible)											
Admission	116	40.1	53*	45.7	36.5–54.9	32	27.6	19.3–35.8	17	14.7	8.1–21.2
Discharge to facility	16	5.5	9	56.3	28.9–83.6	6	37.5	10.9–64.1	5	31.3	5.7–56.8
Discharge home	120	41.5	34	28.3	20.2–36.5	30	25	17.1–32.9	14	11.7	5.8–17.5
Uncertain	22	7.6	8	36.4	14.5–58.2	7	31.8	10.7–53.0	5	22.7	3.7–41.8
Activities of daily living											
Need help with routine tasks	96	33.2	63***	65.6	56.0–75.3	32	33.3	23.7–42.9	26***	27.1	18.0–36.1
Need help with personal care	41	14.2	30***	73.2	59.0–87.3	16	39	23.4–54.6	13**	31.7	16.8–46.6
Need special equipment	117	40.5	57**	48.7	39.5–57.9	39	33.3	24.7–42.0	21	17.9	10.9–25.0
How useful would it be for the ED to offer referrals for…											
Transportation options	196	67.8	66	33.7	27.0–40.4	51	26	19.8–32.2	28	14.3	9.3–19.2
Food assistance	167	57.8	52	31.1	24.0–38.2	41	24.6	18.0–31.2	26	15.6	10.0–21.1
Housing assistance	156	54	57	36.5	28.9–44.2	37	23.5	17.0–30.5	23	14.7	9.1–20.4
Mental health resources	160	55.4	57	35.6	28.1–43.1	44	27.5	20.5–34.5	24	15	9.4–20.6
Volunteer opportunities	138	47.8	52	37.7	29.5–45.9	37	26.8	19.3–34.3	23	16.7	10.4–23.0
Peer companionship programs	123	42.6	42	33.9	25.4–42.3	34	27.4	19.5–35.4	16	12.9	6.9–18.9

*CI,* confidence interval; *ED*, emergency department.

a Percent with positive screen (as defined in “Methods” section).

* P < 0.05;

** P < 0.01;

*** P < 0.001 under unadjusted bivariate analysis using chi-square tests.

**Table 2:** Characteristics associated with Interpersonal Needs Questionnaire factors, based on stepwise regression, controlling for age and gender.

Characteristic	Multivariable odds ratio (95% CI)		

	Perceived burden	Low belonging	Better off gone
Age (years)	0.97 (0.96–1.04)	1.01 (0.97–1.06)	0.99 (0.95–1.03)
Gender (Male)	1.18 (0.67–2.02)	0.84 (0.47–1.48)	1.1 (0.69–2.05)
Hospitalization in past month	2.09 (1.13–3.85)*	-	-
Needs help with routine tasks	5.87 (3.36–10.27)***	-	5.90 (3.26–10.66)***
Does not drive	-	-	1.33 (0.73–2.44)
Does not talk to family regularly			1.50 (0.56–4.05)
Does not see family regularly	-	3.82 (1.74–8.38)**	-
Does not engage community regularly	-	2.50 (1.35–4.64)**	-
Has difficulty affording food	-	2.50 (1.22–5.12)*	-

*CI,* confidence interval.

* P < 0.05;

** P < 0.01;

*** P < 0.001 under multivariate regression.
